# Genome-wide association study revealed novel loci which aggravate asymptomatic hyperuricaemia into gout

**DOI:** 10.1136/annrheumdis-2019-215521

**Published:** 2019-07-08

**Authors:** Yusuke Kawamura, Hirofumi Nakaoka, Akiyoshi Nakayama, Yukinori Okada, Ken Yamamoto, Toshihide Higashino, Masayuki Sakiyama, Toru Shimizu, Hiroshi Ooyama, Keiko Ooyama, Mitsuo Nagase, Yuji Hidaka, Yuko Shirahama, Kazuyoshi Hosomichi, Yuichiro Nishida, Ippei Shimoshikiryo, Asahi Hishida, Sakurako Katsuura-Kamano, Seiko Shimizu, Makoto Kawaguchi, Hirokazu Uemura, Rie Ibusuki, Megumi Hara, Mariko Naito, Mikiya Takao, Mayuko Nakajima, Satoko Iwasawa, Hiroshi Nakashima, Keizo Ohnaka, Takahiro Nakamura, Blanka Stiburkova, Tony R Merriman, Masahiro Nakatochi, Sahoko Ichihara, Mitsuhiro Yokota, Tappei Takada, Tatsuya Saitoh, Yoichiro Kamatani, Atsushi Takahashi, Kokichi Arisawa, Toshiro Takezaki, Keitaro Tanaka, Kenji Wakai, Michiaki Kubo, Tatsuo Hosoya, Kimiyoshi Ichida, Ituro Inoue, Nariyoshi Shinomiya, Hirotaka Matsuo

**Affiliations:** 1 Department of Integrative Physiology and Bio-Nano Medicine, National Defense Medical College, Tokorozawa, Saitama, Japan; 2 Department of General Medicine, National Defense Medical College, Tokorozawa, Saitama, Japan; 3 Division of Human Genetics, Department of Integrated Genetics, National Institute of Genetics, Mishima, Shizuoka, Japan; 4 Medical Squadron, Air Base Group, Western Aircraft Control and Warning Wing, Japan Air Self-Defense Force, Kasuga, Fukuoka, Japan; 5 Department of Statistical Genetics, Osaka University Graduate School of Medicine, Suita, Osaka, Japan; 6 Laboratory for Statistical Analysis, RIKEN Center for Integrative Medical Sciences, Yokohama, Kanagawa, Japan; 7 Laboratory of Statistical Immunology, Immunology Frontier Research Center (WPI-IFReC), Osaka University, Suita, Osaka, Japan; 8 Department of Medical Biochemistry, Kurume University School of Medicine, Kurume, Fukuoka, Japan; 9 Department of Defense Medicine, National Defense Medical College, Tokorozawa, Saitama, Japan; 10 Midorigaoka Hospital, Takatsuki, Osaka, Japan; 11 Kyoto Industrial Health Association, Kyoto, Japan; 12 Ryougoku East Gate Clinic, Tokyo, Japan; 13 Nagase Clinic, Tokyo, Japan; 14 Akasaka Central Clinic, Tokyo, Japan; 15 Department of Bioinformatics and Genomics, Graduate School of Advanced Preventive Medical Sciences, Kanazawa University, Kanazawa, Ishikawa, Japan; 16 Department of Preventive Medicine, Faculty of Medicine, Saga University, Saga, Japan; 17 Department of International Island and Community Medicine, Kagoshima University Graduate School of Medical and Dental Sciences, Kagoshima, Japan; 18 Department of Preventive Medicine, Nagoya University Graduate School of Medicine, Nagoya, Aichi, Japan; 19 Department of Preventive Medicine, Institute of Health Biosciences, the University of Tokushima Graduate School, Tokushima, Japan; 20 Department of Urology, National Defense Medical College, Tokorozawa, Saitama, Japan; 21 Department of Oral Epidemiology, Hiroshima University Graduate School of Biomedical & Health Sciences, Hiroshima, Japan; 22 Department of Surgery, National Defense Medical College, Tokorozawa, Saitama, Japan; 23 Department of Preventive Medicine and Public Health, National Defense Medical College, Tokorozawa, Saitama, Japan; 24 Department of Geriatric Medicine, Graduate School of Medical Sciences, Kyushu University, Fukuoka, Japan; 25 Laboratory for Mathematics, National Defense Medical College, Tokorozawa, Saitama, Japan; 26 Institute of Rheumatology, Prague, Czech Republic; 27 Department of Pediatrics and Adolescent Medicine, First Faculty of Medicine, Charles University and General University Hospital, Prague, Czech Republic; 28 Department of Biochemisty, University of Otago, Dunedin, New Zealand; 29 Data Science Division, Data Coordinating Center, Department of Advanced Medicine, Nagoya University Hospital, Nagoya, Aichi, Japan; 30 Department of Environmental and Preventive Medicine, Jichi Medical University School of Medicine, Shimotsuke, Tochigi, Japan; 31 Department of Genome Science, School of Dentistry, Aichi Gakuin University, Nagoya, Aichi, Japan; 32 Department of Pharmacy, the University of Tokyo Hospital, Tokyo, Japan; 33 Laboratory of Bioresponse Regulation, Graduate School of Pharmaceutical Sciences, Osaka University, Suita, Osaka, Japan; 34 Division of Inflammation Biology, Institute for Enzyme Research, Tokushima University, Tokushima, Japan; 35 Center for Genomic Medicine, Kyoto University Graduate School of Medicine, Kyoto, Japan; 36 Department of Genomic Medicine, Research Institute, National Cerebral and Cardiovascular Center, Suita, Osaka, Japan; 37 RIKEN Center for Integrative Medical Sciences, Yokohama, Kanagawa, Japan; 38 Division of Kidney and Hypertension, Department of Internal Medicine, Jikei University School of Medicine, Minato-ku, Tokyo, Japan; 39 Department of Pathophysiology and Therapy in Chronic Kidney Disease, Jikei University School of Medicine, Minato-ku, Tokyo, Japan; 40 Department of Pathophysiology, Tokyo University of Pharmacy and Life Sciences, Hachioji, Tokyo, Japan

**Keywords:** asymptomatic hyperuricemia, gout, genome-wide association study, uric acid, individually-tailored preemptive medicine

## Abstract

**Objective:**

The first ever genome-wide association study (GWAS) of clinically defined gout cases and asymptomatic hyperuricaemia (AHUA) controls was performed to identify novel gout loci that aggravate AHUA into gout.

**Methods:**

We carried out a GWAS of 945 clinically defined gout cases and 1003 AHUA controls followed by 2 replication studies. In total, 2860 gout cases and 3149 AHUA controls (all Japanese men) were analysed. We also compared the ORs for each locus in the present GWAS (gout vs AHUA) with those in the previous GWAS (gout vs normouricaemia).

**Results:**

This new approach enabled us to identify two novel gout loci (rs7927466 of *CNTN5* and rs9952962 of *MIR302F*) and one suggestive locus (rs12980365 of *ZNF724*) at the genome-wide significance level (p<5.0×10^–^^8^). The present study also identified the loci of *ABCG2*, *ALDH2* and *SLC2A9*. One of them, rs671 of *ALDH2*, was identified as a gout locus by GWAS for the first time. Comparing ORs for each locus in the present versus the previous GWAS revealed three ‘gout vs AHUA GWAS’-specific loci (*CNTN5*, *MIR302F* and *ZNF724*) to be clearly associated with mechanisms of gout development which distinctly differ from the known gout risk loci that basically elevate serum uric acid level.

**Conclusions:**

This meta-analysis is the first to reveal the loci associated with crystal-induced inflammation, the last step in gout development that aggravates AHUA into gout. Our findings should help to elucidate the molecular mechanisms of gout development and assist the prevention of gout attacks in high-risk AHUA individuals.

**Key messeages**

**What is already known about this subject?**

- We and others, in past genome-wide association studies (GWASs) of gout cases and normouricaemia controls, have identified multiple gout risk loci that elevate serum uric acid.

**What does this study add?**

- We performed the first GWAS of clinically defined gout cases and asymptomatic hyperuricaemia (AHUA) controls.
- This new approach identified two novel gout loci and one suggestive locus that aggravate AHUA into gout.

**How might this impact on clinical practice or future developments?**

- This first discovery of ‘AHUA to gout’ loci using a new GWAS strategy will lead to an understanding of why only a proportion of hyperuricaemia cases develop gout.
- These findings will assist physicians to identify, based on individual genetic differences, AHUA cases who need individually tailored pre-emptive medicine for gout.

## Introduction

**Figure 1:**
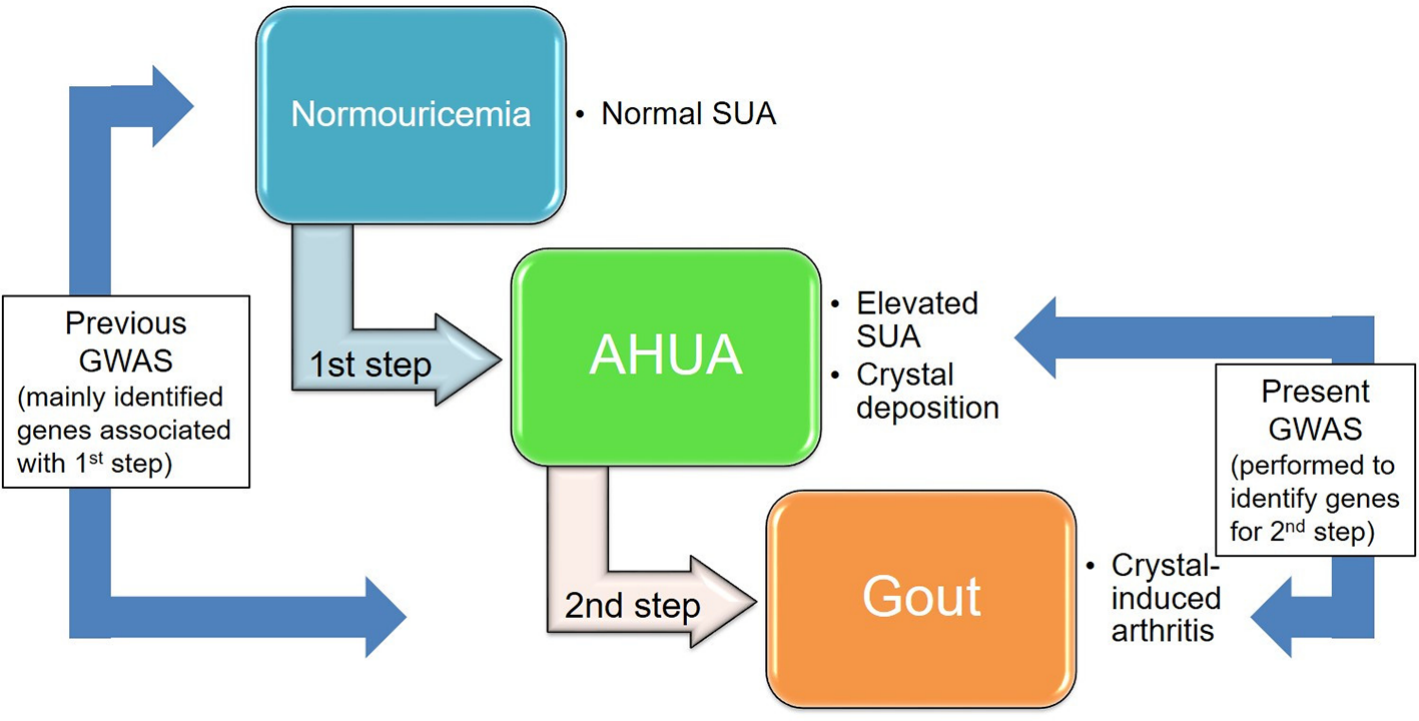
Two steps in the development of gout. We performed, for the first time, a GWAS using clinically defined gout cases and AHUA controls to identify gout loci that influence the progression from hyperuricaemia to gout (the second step). Previous GWASs on gout were performed with gout cases and normouricaemics of which both SUA elevation (the first step) and the second step consisted; however, most of their identified loci were associated with the first step. Because only a proportion of AHUA individuals are known to develop gout, we hypothesised that the genetic effects at the second step would play important roles in crystal-induced inflammation as gout attack. AHUA, asymptomatic hyperuricaemia; GWAS, genome-wide association study; SUA, serum uric acid.

Gout is one of the the most common forms of inflammatory arthritis. It is induced by monosodium urate (MSU) crystals that result from elevated serum uric acid (SUA) level.^1^ The SUA level is determined by the excretion of uric acid via the urate transporters in the kidney and intestine,^2 3^ and the production of uric acid in the liver.^2^ While both genetic and environmental factors are known to cause hyperuricaemia and gout,^4–9^ these diseases are reported to have stronger genetic factors than many other common diseases.^10 11^ Thus far, a number of genes associated with SUA have been identified by genome-wide association studies (GWASs) of SUA,^12–23^ such as the urate transporter genes *ABCG2* (also known as *BCRP*) and *SLC2A9* (also known as *GLUT9*). We and others have performed GWASs of gout by comparing the genetic differences between gout cases and normouricaemia controls^24–26^ and have identified gout risk loci such as *ABCG2* and *SLC2A9*: these are similar results to those from GWASs of SUA. Not all hyperuricaemia cases develop gout: the reason, we consider, is that there are at least two steps by which normouricaemic individuals develop gout. In the first step, the SUA of normouricaemic individuals elevates, creating asymptomatic hyperuricaemia (AHUA); and in the second step, MSU crystal-induced inflammation is experienced as a gout attack (figure 1). Urate transporters such as *ABCG2,* 4 5 27 *SLC2A9,* 28 *SLC22A12* 29 30 and *SLC17A1* 31 naturally play important roles in the first step, but there must be other loci for the second step that aggravates AHUA into a gout attack. In this study, for the first time, we performed a gout GWAS using clinically defined gout cases and AHUA controls to identify risk loci that uniquely influence the progression from AHUA to gout, as distinct from causing SUA elevation.

## Methods

### Study subjects

In the present study, we avoided the use of self-reported gout cases and AHUA controls and collected only clinically defined individuals.

All gout cases were clinically diagnosed with primary gout according to the criteria established by the American College of Rheumatology.^32^ All patients were assigned from Japanese male outpatients at the gout clinics of Midorigaoka Hospital (Osaka, Japan), Kyoto Industrial Health Association (Kyoto, Japan), Ryougoku East Gate Clinic (Tokyo, Japan), Nagase Clinic (Tokyo, Japan), Akasaka Central Clinic (Tokyo, Japan) and Jikei University Hospital (Tokyo, Japan). Patients with inherited metabolic disorders, including Lesch-Nyhan syndrome, were excluded. Finally, 2860 Japanese male gout cases were registered as valid case participants. Of these, 945 cases for GWAS stage were the same patients as reported previously.^24 25^

As AHUA controls, 3149 individuals were assigned from among Japanese men with a high SUA level (>7.0 mg/dL) without a history of gout, who were obtained from BioBank Japan^18 33^ and the Shizuoka, Daiko, Fukuoka, Saga and Kagoshima areas in the Japan Multi-Institutional Collaborative Cohort Study.^34 35^ The details of participants in this study are shown in the online supplementary table S1.

10.1136/annrheumdis-2019-215521.supp1 Supplementary data

### Genotyping and quality control

Genome-wide genotyping was performed using Illumina HumanOmniExpress V.1.0 (Illumina) in 1948 individuals (945 cases and 1003 AHUA controls). The data sets were filtered individually on the basis of single nucleotide polymorphism (SNP) genotype missing call rates (>1%) and the Hardy-Weinberg equilibrium (HWE) in AHUA controls (p<1.0 × 10^–6^). We confirmed that all the subjects showed high genotype call rates (>98%). Pairwise identity by state was evaluated to identify pairs of individuals with cryptic relatedness.^36^ We confirmed that there was no pair showing cryptic relatedness greater than expected for second-degree relatives. We performed principal component analysis including our GWAS data set together with HapMap phase II samples^37 38^ as shown in the online supplementary figure S1, indicating that there are no outliers in our GWAS data. Finally, 569 200 SNPs passed filters for 1948 individuals (945 cases and 1003 controls).

**Figure 2:**
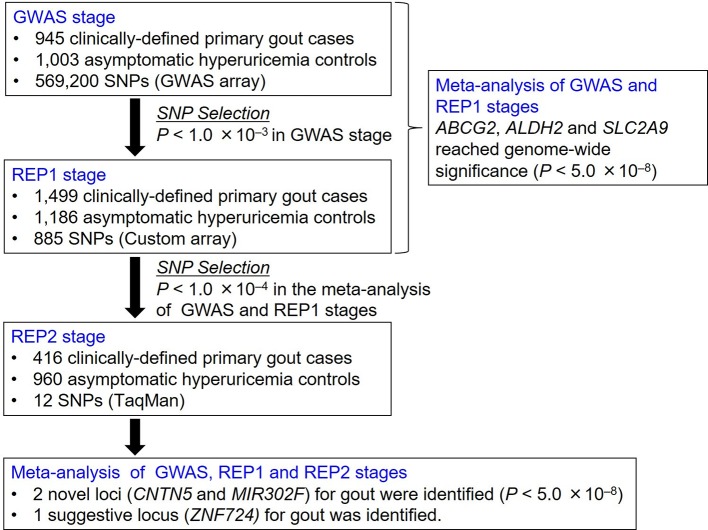
Study design of GWAS of gout cases and asymptomatic hyperuricaemia controls. We performed a GWAS followed by two replication studies (REP1 and REP2 stages) with 2860 Japanese male gout cases and 3149 AHUA controls. Meta-analysis identified two novel loci (*CNTN5* and *MIR302F*) and a suggestive locus (*ZNF724*) at the genome-wide significance level. AHUA, asymptomatic hyperuricaemia; GWAS, genome-wide association study; REP1, the first replication; REP2, the second replication.

At the first replication (REP1) stage, 1246 gout cases and 1186 AHUA controls were genotyped with a custom genotype platform using iSelect HD Custom Genotyping BeadChips (Illumina) on 897 SNPs, and another 253 gout cases were genotyped with Illumina HumanOmniExpress-24 V.1.0 (Illumina). Selected were 897 SNPs using the following criteria: (1) 1000 SNPs were selected as they showed an association (p<0.001 with Fisher’s exact test) in the GWAS stage with gout cases and AHUA controls. (2) After 103 undesignable SNPs had been eliminated, 897 SNPs were selected as the custom genotype platform. For quality control, the data set was filtered individually on the basis of SNP genotype missing call rates (>1%). We excluded subjects with low genotype call rates (<98%). Quality controls for 253 gout cases genotyped with Illumina HumanOmniExpress-24 V.1.0 (Illumina) were performed as described previously.^24^ For REP1 stage, 885 SNPs passed filters for 2685 individuals (1499 cases and 1186 controls) as shown in figure 2.

As the criteria at the second replication (REP2) stage, 68 SNPs passing the significance threshold at p<1.0×10^–4^ in the meta-analysis among the GWAS and REP1 stages were used for the subsequent analyses. In addition to SNPs which had already been reported for gout-associated loci (*ABCG2*, *ALDH2* and *SLC2A9*), we detected top-ranked SNPs among closely located SNPs. We then examined the pairwise linkage disequilibrium (LD) between SNP showing the most significant association and other SNPs. As shown in figure 2, in addition to 3 SNPs of *ABCG2*, *ALDH2* and *SLC2A9*, we finally selected 12 SNPs that were independent of each other at r^2^<0.3 (see online supplementary table S2) for the REP2 stage.

The genotyping for 12 SNPs was performed using an allelic discrimination assay (Custom TaqMan Assay and By-Design, Thermo Fisher Scientific, Waltham, Massachusetts) with a LightCycler 480 (Roche Diagnostics, Mannheim, Germany).^39^ We confirmed that all 12 SNPs were of high call rate (>98%) and HWE in AHUA controls (p>0.001). After quality control, a statistical analysis was performed with 1376 individuals (416 gout cases and 960 AHUA controls). The details of participants in this study are shown in online supplementary table S1.

### Comparison with previous GWAS

The ORs and 95% CIs of gout GWAS with AHUA controls were calculated by meta-analysis with GWAS and REP1 stages in this study, and those of gout GWAS with normouricaemic controls were obtained from our previous study.^25^

### Statistical analysis

We conducted an association analysis using a 2×2 contingency table based on the allele frequency. For each of the filtered SNPs, the p value of association was assessed using Fisher’s exact test, and the OR and 95% CI were calculated. The quantile-quantile plot and the genomic inflation factor (λ) were used to test for the presence of systematic bias in the test statistics due to potential population stratification. The genomic inflation factor (λ) was 1.013, indicating a subtle inflation of p values (see online supplementary figure S2). The results from GWAS, in the first and second replication stages, were combined by meta-analysis.^40^ Inverse-variance fixed-effects model meta-analysis was used for estimating summary OR. Cochran’s Q test^41^ and the I^2^ statistic^42 43^ were examined to assess heterogeneity in ORs among the three studies. If heterogeneity was revealed by statistical testing (p_het_<0.05) or measurement (I^2^ >50%), we implemented a DerSimonian and Laird random-effects model meta-analysis.^44^ All statistical analyses were performed using PLINK V.1.07 and the software R V.3.1.1^45^ with GenABEL and meta packages. The genome-wide significance threshold was set at α=5.0×10^–8^ to reveal any evidence of a significant association.

## Results

### Association analyses

The participants for the GWAS stage were genotyped using HumanOmniExpress V.1.0 (Illumina). Nine hundred forty-five clinically defined gout cases and 1003 AHUA controls passed rigorous quality control filtering (figure 2 and online supplementary figures S1 and S2).

The REP1 stage was then carried out by genotyping 885 SNPs, which showed associations at p<1.0×10^–3^ in the GWAS stage, using a custom genotype platform that employed iSelect HD Custom Genotyping BeadChips (Illumina) in a further 1499 gout cases and 1186 AHUA controls. A meta-analysis was also conducted among the GWAS and REP1 stages (figure 2).

**Figure 3:**
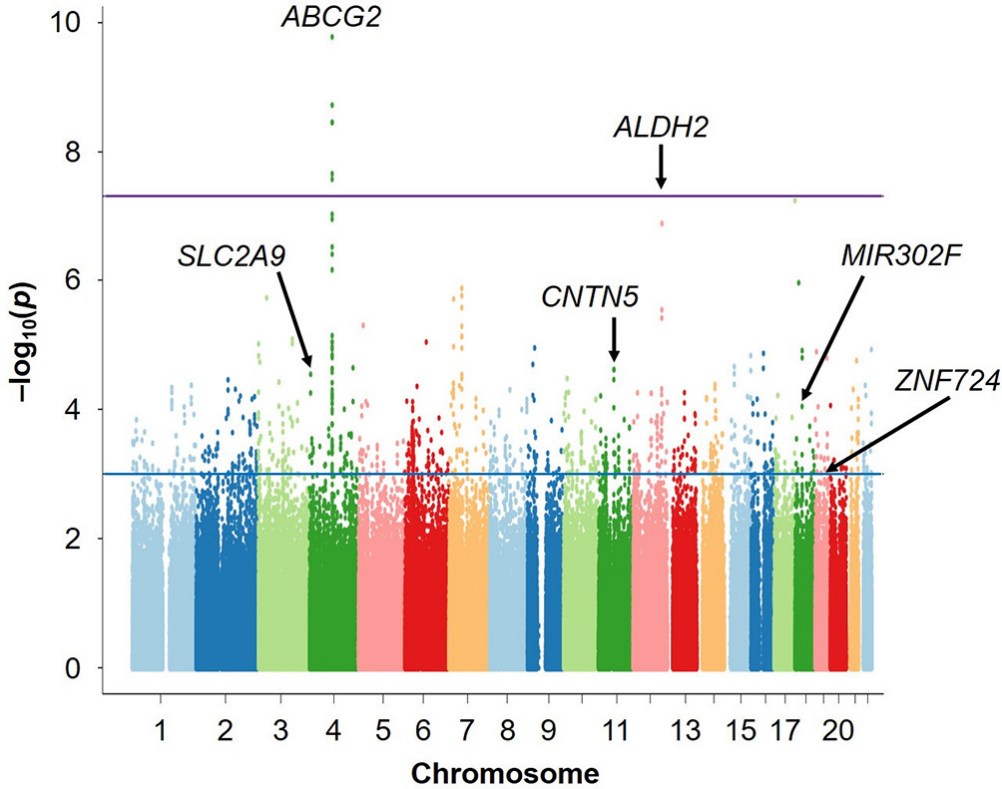
Manhattan plots of the present GWAS (Gout vs AHUA). The x-axis shows chromosomal positions and the y-axis shows −log_10_ p values. The upper purple horizontal line represents the genome-wide significance threshold (p=5.0×10^–8^). The lower blue line indicates the cut-off level for selecting SNPs for REP1 stage (p=1.0×10^–3^). The gene names of identified loci are also shown in the figure. AHUA, asymptomatic hyperuricaemia; GWAS, genome-wide association study; REP1, the first replication; SUA, serum uric acid.

**Figure 4:**
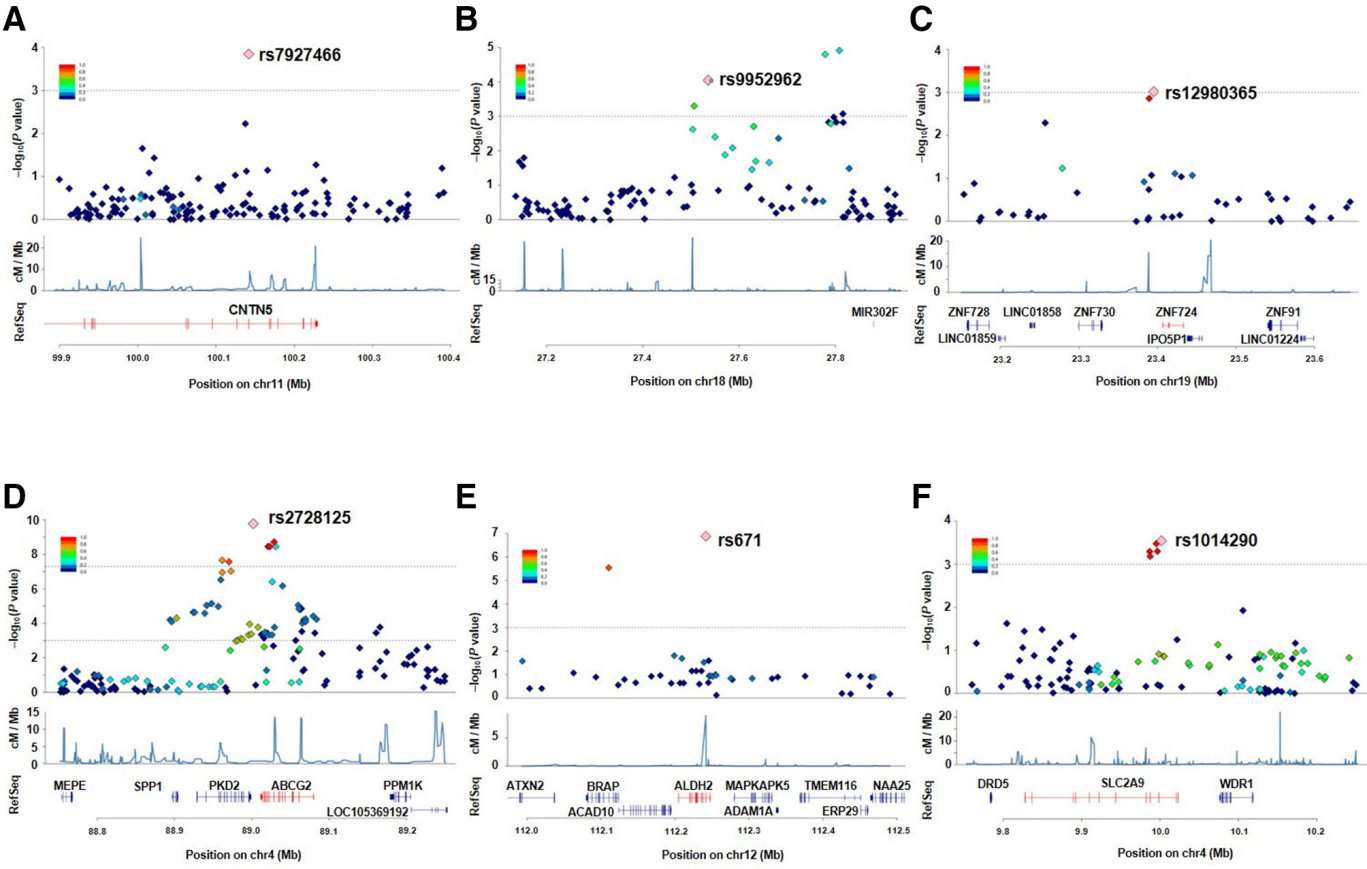
Regional association plots for the six loci identified in the present GWAS (gout vs AHUA). The vertical axis represents -log_10_ (p value) for assessment of the association of each SNP with gout. The highest association signal in each panel is located on (A) *CNTN5*, (B) *MIR302F* as novel loci and (C) *ZNF724* as a suggestive locus and (D) *ABCG2*, (E) *ALDH2* and (F) *SLC2A9* as known loci. The region within 250 kb from the SNP indicating the lowest p value is shown. Top panel: plots of −log_10_ p values for the test of SNP association with gout in the GWAS stage. The SNP showing the lowest p value in the meta-analysis is depicted as a pink diamond. Other SNPs are colour coded according to the extent of linkage disequilibrium (measured in r^2^) with the SNP showing the lowest p value. Middle panel: recombination rates (centimorgans per MB) estimated from HapMap phase II data are plotted. Bottom panel: RefSeq genes. Genomic coordinates are based on NCBI human genome reference sequence build 37. Details of the results for the six loci are also shown in table 1. AHUA, asymptomatic hyperuricaemia; GWAS, genome-wide association study; NCBI, National Center for Biotechnology Information; SNP, single nucleotide polymorphism.

As a result, we identified three loci showing the associations at the genome-wide significance level (p<5.0×10^–8^): rs2728125 of *ABCG2* (p=6.58×10^–20^; OR=0.67), rs671 of *ALDH2* (p=4.44×10^–14^; OR=0.68) and rs1014290 of *SLC2A9* (p=2.29×10^–9^; OR=1.30; figures 3 and 4, table 1). Of these, the loci of *ABCG2* and *SLC2A9* were also detected in the previous GWAS of gout cases and normouricaemia controls.^24^ rs671 of *ALDH2* was identified as a gout-associated SNP in a subsequent fine mapping study.^46^ The present study therefore identified rs671 of *ALDH2* as a gout locus by GWAS for the first time.

**Table 1:** Five SNPs showing significant association at the genome-wide significance level and one suggestive SNP

	Allele*				GWAS stage‡				REP1 stage§				REP2 stage¶				Meta-analysis**					
					Risk allele frequency				Risk allele frequency				Risk allele frequency				Fixed-effect model		Random-effect model		Cochran's Q	
SNP	A/B	Chr	Position†	Gene	Case	Control	OR (95% CI)	P value	Case	Control	OR (95% CI)	P value	Case	Control	OR (95% CI)	P value	OR (95% CI)	P value	OR (95% CI)	P value	P value	*I* ^2^
rs2728125	G/A	4	89 001 893	*ABCG2*	0.401	0.304	1.54 (1.35 to 1.76)	1.76×10^−10^	0.400	0.313	1.46 (1.31 to 1.64)	5.33×10^−11^	NA	NA	NA	NA	1.49 (1.37 to 1.63)	6.58×10^−20^	1.49 (1.37 to 1.63)	6.58×10^−20^	0.57	0
rs671	C/T	12	112 241 766	*ALDH2*	0.821	0.751	1.52 (1.30 to 1.77)	1.33×10^−7^	0.821	0.760	1.44 (1.26 to 1.65)	5.94×10^−8^	NA	NA	NA	NA	1.47 (1.33 to 1.63)	4.44×10^−14^	1.47 (1.33 to 1.63)	4.44×10^−14^	0.62	0
rs1014290	T/C	4	10 001 861	*SLC2A9*	0.678	0.623	1.28 (1.12 to 1.46)	2.75×10^−4^	0.674	0.611	1.31 (1.17 to 1.47)	2.03×10^−6^	NA	NA	NA	NA	1.30 (1.19 to1.41)	2.29×10^−9^	1.30 (1.19 to 1.41)	2.29×10^−9^	0.76	0
rs7927466	A/G	11	100 141 763	*CNTN5*	0.976	0.953	1.99 (1.39 to 2.85)	1.59×10^−4^	0.973	0.954	1.74 (1.30 to 2.33)	2.29×10^−4^	0.976	0.954	1.91 (1.16 to 3.12)	1.03×10^−2^	1.85 (1.50 to 2.27)	5.33×10^−9^	1.85 (1.50 to 2.27)	5.33×10^−9^	0.84	0
rs9952962	C/T	18	27 535 568	*MIR302F*	0.549	0.486	1.29 (1.14 to 1.46)	8.07×10^−5^	0.547	0.506	1.18 (1.06 to 1.31)	2.97×10^−3^	0.552	0.490	1.28 (1.09 to 1.51)	2.92×10^−3^	1.24 (1.15 to 1.33)	1.67×10^−8^	1.24 (1.15 to 1.33)	1.67×10^−8^	0.50	0
rs12980365	A/G	19	23 395 317	*ZNF724*	0.976	0.957	1.84 (1.27 to 2.65)	1.12×10^−3^	0.974	0.959	1.61 (1.19 to 2.18)	1.97×10^−3^	0.975	0.948	2.09 (1.29 to 3.36)	2.58×10^−3^	1.77 (1.43 to 2.18)	9.76×10^−8^	1.77 (1.43 to 2.18)	9.76×10^−8^	0.65	0

*Allele A is risk-associated allele, and allele B is non-risk-associated allele.

†SNP positions are based on NCBI human genome reference sequence Build 37.4.

‡945 gout cases and 1003 AHUA controls.

§1499 gout cases and 1186 AHUA controls.

¶416 gout cases and 960 AHUA controls.

**Meta-analyses of the combined GWAS and replication samples (2860 gout cases and 3149 controls of Japanese men).

AHUA, asymptomatic hyperuricaemia; Chr, Chromosome; GWAS, genome-wide association study;NCBI, National Center for Biotechnology Information; REP1, the first replication; REP2, the second replication;SNP, single nucleotide polymorphism.

To identify additional risk loci, we recruited independent participants comprising 416 gout cases and 960 AHUA controls. Twelve SNPs were selected for the REP2 stage by considering LD among 68 SNPs showing associations at p<1.0×10^–4^ in the meta-analysis among the GWAS and REP1 stages. Genotyping of these 12 SNPs was performed by TaqMan assay, and the meta-analysis was conducted among the GWAS, REP1 and REP2 stages (figure 2). Online supplementary tables S2A and S2B summarise the GWAS and replication study of three SNPs which have been reported to have gout-associated loci (*ABCG2*, *ALDH2* and *SLC2A9*) and the 12 SNPs selected for the REP2 stage.

Finally, two novel loci achieved genome-wide significance in the meta-analysis of three stages (figures 3 and 4, table 1): an intronic SNP of *CNTN5*, rs7927466 (p_meta_=5.33×10^–9^; OR=1.85) and an intergenic SNP located on near *MIR302F*, rs9952962 (p_meta_=1.67×10^–8^; OR=0.81). In addition, an intergenic SNP nearing *ZNF724* (rs12980365) showed a suggestive level of association (p_meta_=9.76×10^–8^; OR=1.77).

### Comparison with previous GWASs

**Figure 5:**
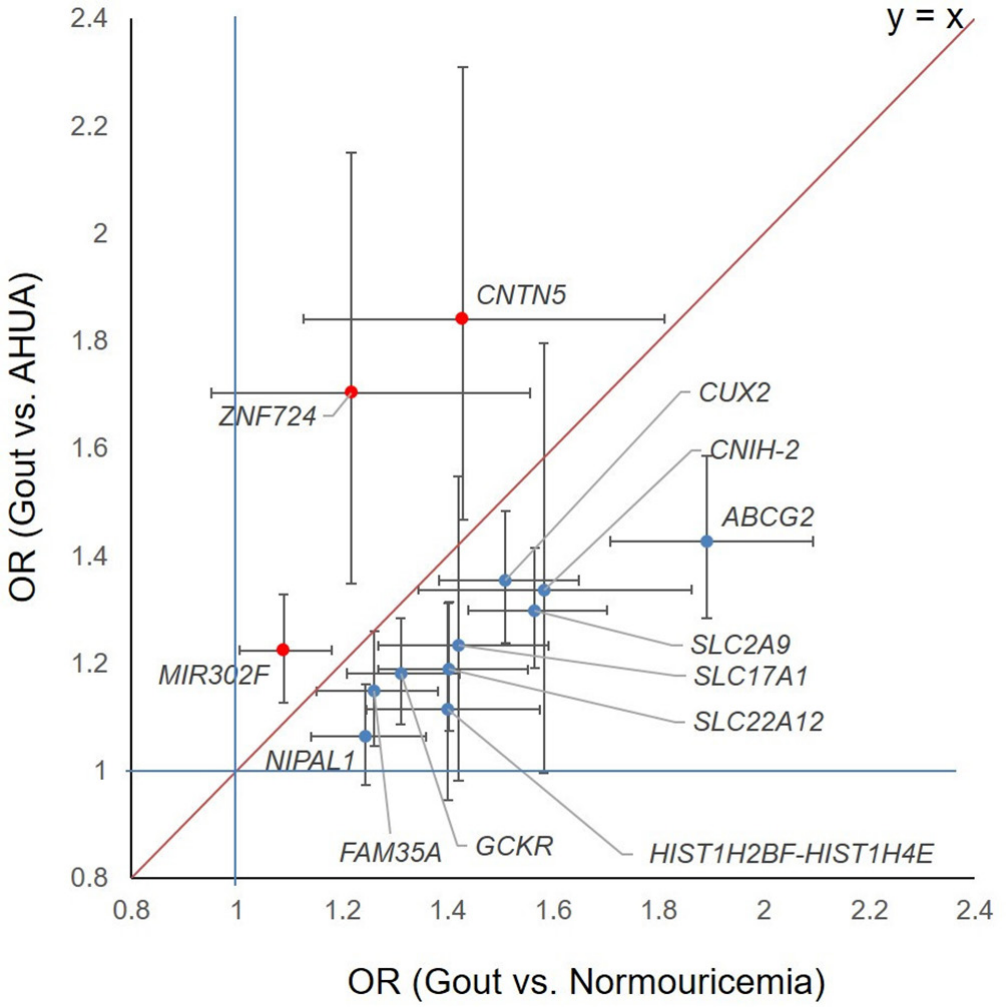
Comparison ORs for three gout loci by the present GWAS (gout vs AHUA) with ORs for 10 gout loci by the previous GWAS (gout vs normouricaemia). Dots represent ORs and lines represent 95% CIs. The horizontal axis shows ORs for gout compared with normouricaemia, and the vertical axis shows ORs for gout compared with AHUA, whereas 10 ’normouricaemia to gout’ loci are located under the oblique line, three novel ‘AHUA to gout’ loci are above it. AHUA, asymptomatic hyperuricaemia; GWAS, genome-wide association study.

We investigated whether or not these identified risk loci are associated with gout susceptibility via SUA elevation (the first step in figure 1). We compared the ORs for each locus in the present GWAS (gout vs AHUA) with those in the previous GWAS^25^ (gout vs normouricaemia): that is, the 3 loci identified in this study (‘AHUA to gout’ loci; *CNTN5*, *MIR302F* and *ZNF724*) and 10 previously identified risk loci^25^ (‘normouricaemia to gout’ loci; *ABCG2*, *SLC2A9*, *CUX2*, *SLC22A12*, *GCKR*, *SLC17A1*, *HIST1H2BF-HIST1H4E*, *CNIH-2*, *NIPAL1* and *FAM35A*) (figure 5 and online supplementary table S3). Interestingly, when plotted, the ‘AHUA to gout’ loci and ‘normouricaemia to gout’ loci appeared as distinct patterns (figure 5). The ‘normouricaemia to gout’ loci trended under the oblique line, whereas all three ‘AHUA to gout’ loci were located above the oblique line, clearly indicating that these novel loci are associated with distinct mechanisms of gout development that differs from those of 10 known gout loci (see online supplementary table S3).^25^ It is consistent with the finding that ‘the ratio of the two ORs’ of each locus in the present GWAS (gout vs AHUA) is >1, whereas that of each locus in the previous GWAS (gout vs normouricaemia) is <1 (see online supplementary table S3).^25^ We also investigated the effect of each locus on SUA using the results from our recent GWAS meta-analysis of SUA with a total of 121 745 Japanese subjects^47^ and the results from GWAS meta-analysis of SUA with a total of 110 347 individuals of European ancestry within the Global Urate Genetics Consortium (GUGC).^22^ The association results for each locus are shown in online supplementary table S4. Both results are consistent with those of the present study (shown in figure 5). Furthermore, we also investigated the association results for three gout locus identified in the present GWAS from the result of the gout GWAS (gout vs non-gout) using a total of 69 374 individuals (2115 gout cases and 67 259 controls) of European ancestry within GUGC (see online supplementary table S5).^22^ The two SNPs that were polymorphic in European populations were evaluated. After Bonferroni correction (p<2.5×10^–2^=0.05/2), rs12980365 of *ZNF724* showed a significant association with gout in persons of European ancestry (p=8.54 × 10^–3^, online supplementary table S5).

## Discussion

It is well known that gout has stronger genetic contributors than other common diseases, and that only a proportion of AHUA individuals develop gout, but no studies have hitherto reported the differences in genetic background between gout and AHUA. Through the present study, we first performed GWAS with gout cases and AHUA controls and identified two novel loci that have a stronger effect on the second step (AHUA to gout) in the development of gout. The present study detected the loci of *ABCG2*, *SLC2A9* and *ALDH2*. One of them, rs671 of *ALDH2*, was identified as a gout locus by GWAS for the first time.

We have also discovered that rs7927466 of *CNTN5* is a gout susceptibility locus. *CNTN5* is a member of the contactin family, which mediates cell surface interactions during the development of the nervous system.^48 49^ There are several reports that *CNTN5* is associated with neuropsychiatric disorders, such as autism spectrum disorder,^50 51^ attention deficit hyperactivity disorder^52^ and anorexia nervosa.^53^ Interestingly, *CNTN5* has also been reported to be associated with inflammatory diseases including ankylosing spondylitis^54^ and Behçet disease.^55^ It has also been reported that another intronic SNP of *CNTN5* (rs1813445) is associated with the response to anti-tumour necrosis factor therapy in rheumatoid arthritis^56^ and Crohn's disease.^57^ Since the present findings indicate that the SNPs of *CNTN5* could be involved in the second step of gout development, it is assumed that *CNTN5* polymorphisms could also cause inflammation at the joints where MSU crystals are deposited resulting from high SUA.

We also identified rs9952962, an SNP near *MIR302F* as a novel gout locus. MicroRNAs (miRNAs) were discovered in 1993^58^: they are small non-coding RNA molecules which play an important role in regulating gene expression.^59^ It is reported that miR-302f is deregulated in gastric cancer^60^ and that chemotherapy modifies miR-302f expression in esophageal cancer.^61^ As several miRNAs have been identified as being involved in the pathogenesis of other non-infectious forms of inflammatory arthritis, including rheumatoid arthritis,^62 63^ some reports have also shown a relationship between miRNA and gouty arthritis.^64–66^ Our results might suggest that miR-302f affects the inflammation seen in gouty arthritis by modulating gene expression, although further analysis will be needed to elucidate the relationship between them.

We also identified rs12980365, an SNP of *ZNF724*, as a potential gout locus. However, there are no reports so far on *ZNF724*. It is of course possible that the identified loci in the present study are just surrogate markers and that other genes including *ZNF730* and *IPO5P1* near these SNPs are the true risk loci for gout development. The limitation is the lack of imputation because we applied the study design shown in figure 2. Further analysis of larger samples with imputation will be the key to identifying more gout loci.

The unique finding in the present study is that the novel loci for ‘AHUA to gout’ and known loci for ‘normouricaemia to gout’ appear to relate to different molecular mechanisms of gout development (figure 5 and online supplementary table S3). The ‘AHUA to gout’ loci identified in the present study appear to be involved in the second step of development from AHUA to gouty arthritis rather than the ‘normouricaemia to gout’ loci (figure 1). Since only a proportion of hyperuricaemia cases develop gout and most hyperuricaemia cases remain as AHUA cases, the molecules involved in this step are likely to play important roles in innate immunity and/or inflammation in response to MSU deposition.

The frequency of female gout cases is low (1.15% in our data) in Japan, which shows that analysing only male gout patients in the present study should be more appropriate for detecting genetic factors of gout. Because each locus identified in the present GWAS did not show a significant association with SUA in persons of European ancestry or in Japanese individuals (see online supplementary table S4), our findings show that they are not likely to be a locus that is associated with AHUA susceptibility. Interestingly, the present study also demonstrated that rs12980365 of *ZNF724* showed a significant association with gout in persons of European ancestry within GUGC, which compared gout and non-gout individuals. This finding suggests that *ZNF724* is a novel gout locus that aggravates AHUA into gout, also in individuals of European ancestry. However, since the GWAS within the GUGC was a study using non-gout individuals as controls, it is necessary to perform replication studies using AHUA as controls. Thus, further analyses of independent populations using AHUA controls will be required in the future (see online supplementary table S5).

This first discovery of ‘AHUA to gout’ loci using a new GWAS strategy will lead to elucidation of the molecular mechanism of the last step of gout development, which will clarify the individual genetic differences that explain why only a proportion of hyperuricaemia cases develop gout and to the prevention of gout attacks in high-risk AHUA individuals. These findings will assist physicians to identify AHUA cases who need adequate preemptive medicine for gout based on individual genetic differences.http://dx.doi.org/10.1136/annrheumdis-2019-215521
