# Supplementary material for: Genome-wide association study revealed novel loci which aggravate asymptomatic hyperuricaemia into gout
Source: Ann Rheum Dis. 2019 Jul 8;78(10):1430–7. doi: 10.1136/annrheumdis-2019-215521 (PMC6788923; doi:10.1136/annrheumdis-2019-215521)
Supplement: Supplementary data [file annrheumdis-2019-215521supp001.pdf]

Supplementary Materials

A genome-wide association study revealed novel loci which aggravate asymptomatic hyperuricemia into gout

Yusuke Kawamura, Hirofumi Nakaoka, Akiyoshi Nakayama, Yukinori Okada,
Ken Yamamoto *et al.*

Correspondence to Matsuo H (e-mail: hmatsuo@ndmc.ac.jp).

Supplementary Figure S1 Results of principal component analysis including case and control samples used in the present GWAS and four HapMap populations

Supplementary Figure S2 Quantile-quantile plot of *P* value distribution for association

Supplementary Table S1 Characteristics of participants

Supplementary Table S2 Summary of GWAS and replication study of 13 SNPs

Supplementary Table S3 The effect of each gout locus according to the present GWAS (Gout vs. AHUA) and the previous GWAS (Gout vs. Normouricemia)

Supplementary Table S4 The effect of each gout locus on SUA

Supplementary Table S5 The effect of each gout locus from the results of the gout GWAS (Gout vs. Non-gout) using individuals of European ancestry

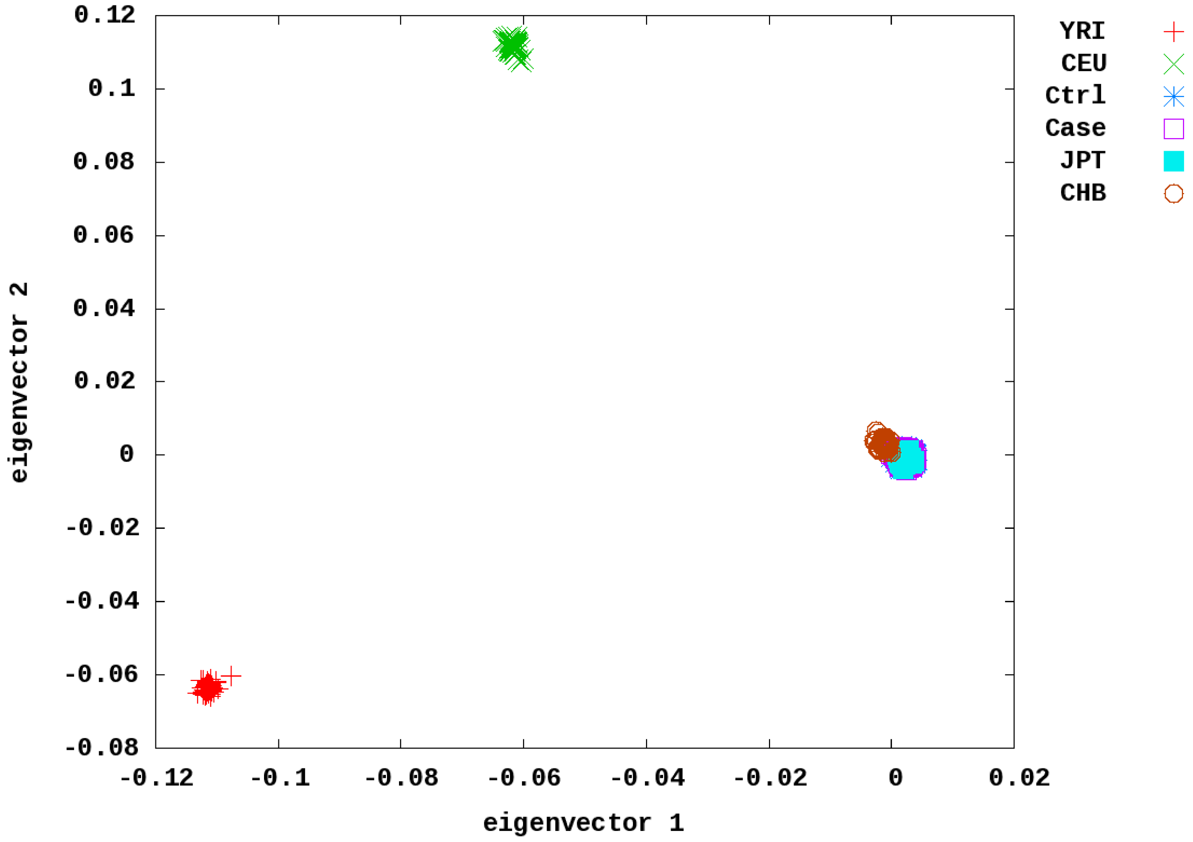

Supplementary Figure S1 Results of principal component analysis including case and control samples used in the present GWAS and four HapMap populations.

JPT, Japanese in Tokyo, Japan. CEU, Utah residents with Northern and Western European ancestry from the CEPH collection. YRI, Yoruba in Ibadan, Nigeria. CHB, Han Chinese in Beijing, China. GWAS, genome-wide association study.

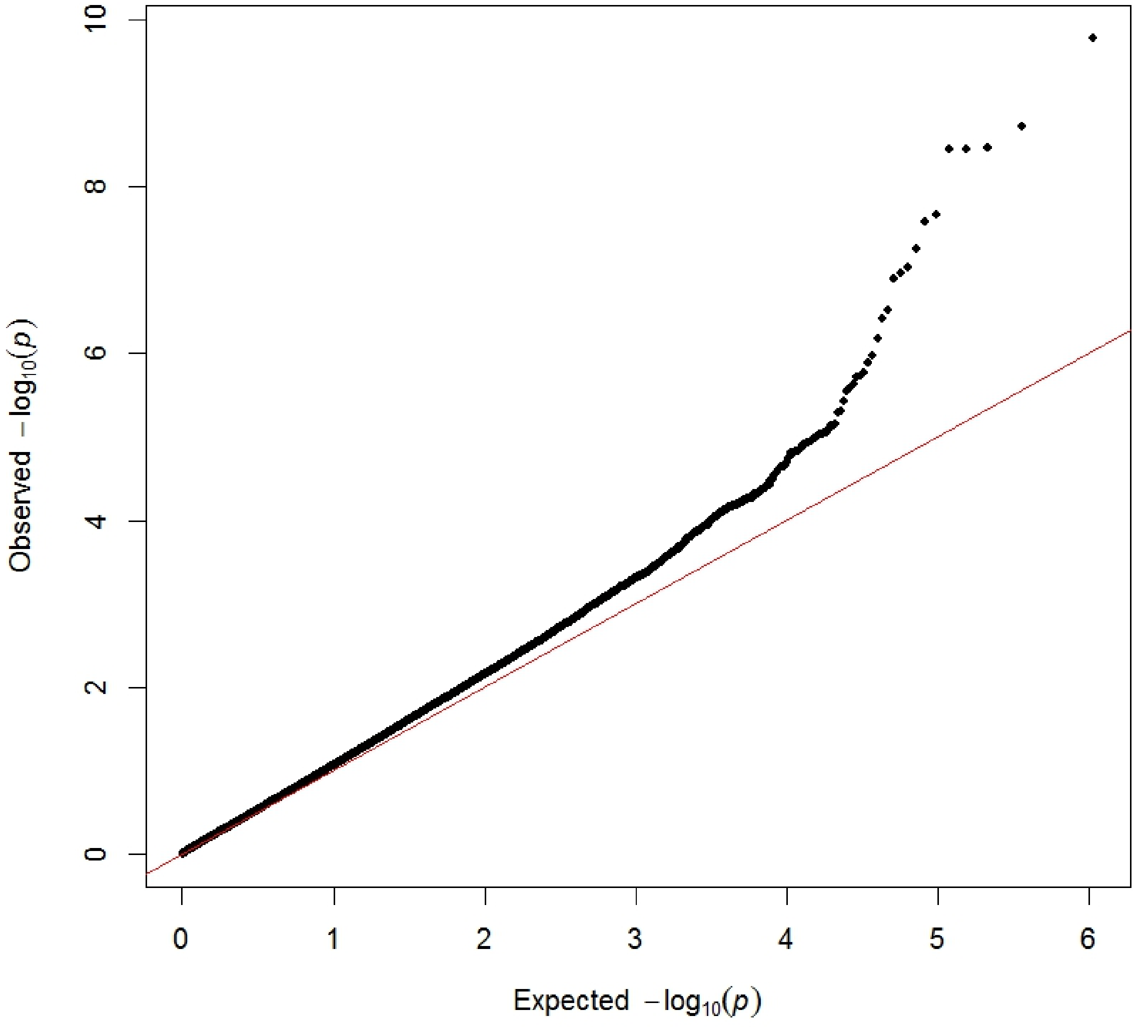

Supplementary Figure S2 Quantile-quantile plot of *P* value distribution for association.
The results are plotted as black dots. The genomic inflation factor (λ) was 1.013.

Supplementary Table S1 Characteristics of participants.

	GWAS stage		REP1 stage		REP2 stage	
	Gout case	AHUA control	Gout case	AHUA control	Gout case	AHUA control
Number	945	1,003	1,499	1,186	416	960
Age (years)	46.6 ± 11.3	57.0 ± 15.0	45.1 ± 11.5	56.4 ± 9.1	52.3 ± 11.8	55.2 ± 9.1
Body mass index (kg/m^2)	25.1 ± 3.6	23.6 ± 3.8	24.9 ± 3.4	24.4 ± 3.0	24.9 ± 3.5	24.9 ± 3.2

Plus-minus values are means ± SD. All selected participants were Japanese males.

GWAS, genome-wide association study; REP1, the first replication; REP2, the second replication; AHUA, asymptomatic hyperuricemia

Supplementary Table S2A Summary of GWAS and replication study of 15 SNPs

					GWAS stage‡				REP1 stage§				REP2 stage¶			
					Risk Allele frequency				Risk Allele frequency				Risk Allele frequency			
SNP	Allele* A/B	Chr	Position†	Gene	Case	Control	OR (95%CI)	P value	Case	Control	OR (95%CI)	P value	Case	Control	OR (95%CI)	P value
rs2728125	G/A	4	89001893	*ABCG2*	0.401	0.304	1.54 (1.35-1.76)	1.76×10^{-10}	0.400	0.313	1.46 (1.31-1.64)	5.33×10^{-11}	NA	NA	NA	NA
rs671	C/T	12	112241766	*ALDH2*	0.821	0.751	1.52 (1.30-1.77)	1.33×10^{-7}	0.821	0.760	1.44 (1.26-1.65)	5.94×10^{-8}	NA	NA	NA	NA
rs1014290	T/C	4	10001861	*SLC2A9*	0.678	0.623	1.28 (1.12-1.46)	2.75×10^{-4}	0.674	0.611	1.31 (1.17-1.47)	2.03×10^{-6}	NA	NA	NA	NA
rs7927466	A/G	11	100141763	*CNTN5*	0.976	0.953	1.99 (1.39-2.85)	1.59×10^{-4}	0.973	0.954	1.74 (1.30-2.33)	2.29×10^{-4}	0.976	0.954	1.91 (1.16-3.12)	1.03×10^{-2}
rs10764052	C/T	10	19823467	*C10orf112*	0.577	0.521	1.25 (1.10-1.42)	4.64×10^{-4}	0.554	0.509	1.20 (1.08-1.34)	9.02×10^{-4}	0.522	0.506	1.07 (0.91-1.26)	0.441
rs9952962	C/T	18	27535568	*MIR302F*	0.549	0.486	1.29 (1.14-1.46)	8.07×10^{-5}	0.547	0.506	1.18 (1.06-1.31)	2.97×10^{-3}	0.552	0.490	1.28 (1.09-1.51)	2.92×10^{-3}
rs430312	A/G	3	8646024	*C3orf32*	0.817	0.774	1.31 (1.12-1.53)	8.36×10^{-4}	0.827	0.796	1.22 (1.06-1.40)	4.88×10^{-3}	0.820	0.797	1.17 (0.95-1.44)	0.149
rs12980365	A/G	19	23395317	*ZNF724*	0.976	0.957	1.84 (1.27-2.65)	1.12×10^{-3}	0.974	0.959	1.61 (1.19-2.18)	1.97×10^{-3}	0.975	0.948	2.09 (1.29-3.36)	2.58×10^{-3}
rs1113976	G/A	10	69517922	*TRNAS21*	0.528	0.469	1.27 (1.12-1.44)	2.10×10^{-4}	0.520	0.485	1.15 (1.03-1.28)	1.14×10^{-2}	0.507	0.491	1.07 (0.91-1.26)	0.438
rs11942233	C/T	4	1634506	*FAM53A*	0.079	0.051	1.58 (1.22-2.05)	5.20×10^{-4}	0.071	0.055	1.32 (1.05-1.65)	1.59×10^{-2}	0.084	0.056	1.55 (1.13-2.12)	6.19×10^{-3}
rs11070570	A/G	15	47426957	*SEMA6D*	0.714	0.662	1.28 (1.11-1.46)	4.47×10^{-4}	0.703	0.674	1.15 (1.02-1.29)	2.20×10^{-2}	0.696	0.673	1.12 (0.94-1.33)	0.224
rs3107329	A/G	3	151624629	*SUCNR1*	0.901	0.859	1.48 (1.22-1.81)	8.23×10^{-5}	0.891	0.868	1.24 (1.05-1.47)	1.01×10^{-2}	0.875	0.848	1.26 (0.99-1.61)	6.03×10^{-2}
rs6801125	C/A	3	94055820	*LOC389137*	0.850	0.803	1.39 (1.18-1.65)	1.08×10^{-4}	0.832	0.805	1.20 (1.04-1.38)	1.16×10^{-2}	0.831	0.800	1.23 (0.99-1.52)	6.23×10^{-2}
rs9294467	A/C	6	91517782	*MAP3K7*	0.312	0.263	1.27 (1.11-1.46)	6.53×10^{-4}	0.296	0.269	1.15 (1.02-1.29)	2.65×10^{-2}	0.265	0.256	1.05 (0.87-1.26)	0.631
rs4418540	A/G	1	80586000	*LOC553139*	0.600	0.546	1.25 (1.10-1.41)	7.24×10^{-4}	0.583	0.551	1.14 (1.02-1.27)	2.02×10^{-2}	0.584	0.545	1.17 (0.99-1.38)	6.32×10^{-2}

*Allele A is risk-associated allele and allele B is non-risk-associated allele.

†SNP positions are based on NCBI human genome reference sequence Build 37.4

‡945 gout cases and 1,003 AHUA controls

§1,499 gout cases and 1,186 AHUA controls

¶416 gout cases and 960 AHUA controls

**Meta-analyses of the combined GWAS and replication samples (2,860 gout cases and 3,149 controls of Japanese male)

SUA, serum uric acid; GWAS, genome-wide association study; REP1, the first replication; REP2, the second replication; Chr, Chromosome; AHUA, asymptomatic hyperuricemia.

Supplementary Table S2B Summary of GWAS and replication study of 15 SNPs

					Meta-analysis**					
					Fixed effect model		Random effect model		Cochran's Q	
SNP	Allele* A/B	Chr	Position†	Gene	OR (95%CI)	P value	OR (95%CI)	P value	P value	I^2
rs2728125	G/A	4	89001893	*ABCG2*	1.49 (1.37-1.63)	6.58×10^{-20}	1.49 (1.37-1.63)	6.58×10^{-20}	0.57	0
rs671	C/T	12	112241766	*ALDH2*	1.47 (1.33-1.63)	4.44×10^{-14}	1.47 (1.33-1.63)	4.44×10^{-14}	0.62	0
rs1014290	T/C	4	10001861	*SLC2A9*	1.30 (1.19-1.41)	2.29×10^{-9}	1.30 (1.19-1.41)	2.29×10^{-9}	0.76	0
rs7927466	A/G	11	100141763	*CNTN5*	1.85 (1.50-2.27)	5.33×10^{-9}	1.85 (1.50-2.27)	5.33×10^{-9}	0.84	0
rs10764052	C/T	10	19823467	*C10orf112*	1.19 (1.11-1.28)	3.60×10^{-6}	1.19 (1.09-1.29)	3.63×10^{-5}	0.30	0.17
rs9952962	C/T	18	27535568	*MIR302F*	1.24 (1.15-1.33)	1.67×10^{-8}	1.24 (1.15-1.33)	1.67×10^{-8}	0.50	0
rs430312	A/G	3	8646024	*C3orf32*	1.24 (1.13-1.36)	6.44×10^{-6}	1.24 (1.13-1.36)	6.44×10^{-6}	0.67	0
rs12980365	A/G	19	23395317	*ZNF724*	1.77 (1.43-2.18)	9.76×10^{-8}	1.77 (1.43-2.18)	9.76×10^{-8}	0.65	0
rs1113976	G/A	10	69517922	*TRNAS21*	1.17 (1.09-1.26)	2.44×10^{-5}	1.17 (1.07-1.28)	7.52×10^{-4}	0.23	0.32
rs11942233	C/T	4	1634506	*FAM53A*	1.45 (1.25-1.69)	9.35×10^{-7}	1.45 (1.25-1.69)	9.35×10^{-7}	0.52	0
rs11070570	A/G	15	47426957	*SEMA6D*	1.18 (1.09-1.28)	3.47×10^{-5}	1.18 (1.09-1.28)	3.47×10^{-5}	0.39	0
rs3107329	A/G	3	151624629	*SUCNR1*	1.32 (1.18-1.48)	1.16×10^{-6}	1.32 (1.18-1.48)	1.26×10^{-6}	0.37	6.0×10^{-3}
rs6801125	C/A	3	94055820	*LOC389137*	1.26 (1.15-1.39)	1.72×10^{-6}	1.26 (1.15-1.39)	1.72×10^{-6}	0.38	0
rs9294467	A/C	6	91517782	*MAP3K7*	1.17 (1.08-1.27)	1.97×10^{-4}	1.16 (1.05-1.29)	3.06×10^{-3}	0.23	0.31
rs4418540	A/G	1	80586000	*LOC553139*	1.18 (1.10-1.27)	1.22×10^{-5}	1.18 (1.10-1.27)	1.22×10^{-5}	0.57	0

*Allele A is risk-associated allele and allele B is non-risk-associated allele.

†SNP positions are based on NCBI human genome reference sequence Build 37.4

‡945 gout cases and 1,003 AHUA controls

§1,499 gout cases and 1,186 AHUA controls

¶416 gout cases and 960 AHUA controls

**Meta-analyses of the combined GWAS and replication samples (2,860 gout cases and 3,149 controls of Japanese male)

SUA, serum uric acid; GWAS, genome-wide association study; REP1, the first replication; REP2, the second replication; Chr, Chromosome; AHUA, asymptomatic hyperuricemia.

Supplementary Table S3 The effect of each gout locus according to the present GWAS (Gout vs. AHUA) and the previous GWAS (Gout vs. Normouricemia)

SNP	Allele* A/B	Locus	Chr	Position	Gene	Gout vs. AHUA†		Gout vs. Normouricemia‡		Ratio of two ORs§
						OR (95%CI)	P value	OR (95%CI)	P value	
Gout loci identified in the present GWAS (Gout vs. AHUA)										
rs7927466	A/G	11q22.1	11	100141763	*CNTN5*	1.84 (1.47-2.31)	1.38×10^{-7}	1.43 (1.13-1.81)	3.28×10^{-3}	1.29
rs9952962	C/T	18q12.1	18	27535568	*MIR302F*	1.22 (1.13-1.33)	1.42×10^{-6}	1.09 (1.00-1.18)	3.86×10^{-2}	1.12
rs12980365	A/G	19p12	19	23395317	*ZNF724*	1.70 (1.35-2.15)	7.44×10^{-6}	1.22 (0.95-1.56)	0.117	1.40
Gout loci reported in the previous GWAS (Gout vs. Normouricemia)‡										
rs1260326	T/C	2p23.3	2	27730940	*GCKR*	1.18 (1.09-1.28)	8.99×10^{-5}	1.31 (1.21-1.42)	7.19×10^{-11}	0.901
rs1014290	T/C	4p16.1	4	10001861	*SLC2A9*	1.30 (1.19-1.41)	2.31×10^{-9}	1.56 (1.44-1.70)	6.50×10^{-26}	0.830
rs11733284	A/G	4p12	4	48028097	*NIPAL1*	1.06 (0.97-1.16)	0.171	1.24 (1.14-1.36)	9.05×10^{-7}	0.854
rs3114020	C/T	4q22.1	4	89083666	*ABCG2*	1.43 (1.28-1.59)	5.25×10^{-11}	1.89 (1.70-2.09)	8.66×10^{-35}	0.754
rs1165176	G/A	6p22.2	6	25830298	*SLC17A1*	1.23 (0.98-1.55)	7.08×10^{-2}	1.42 (1.27-1.59)	1.47×10^{-9}	0.868
rs11758351	G/T	6p22.2	6	26203910	*HIST1H4E*	1.11 (0.95-1.31)	0.197	1.40 (1.25-1.57)	1.63×10^{-8}	0.795
rs7903456	A/G	10q23.2	10	88919319	*FAM35A*	1.15 (1.05-1.26)	3.35×10^{-3}	1.26 (1.15-1.38)	6.45×10^{-7}	0.911
rs2285340	A/G	11q13.1	11	64435906	*SLC22A12*	1.19 (1.07-1.31)	7.72×10^{-4}	1.40 (1.27-1.55)	4.61×10^{-11}	0.847
rs4073582	C/T	11q13.2	11	66050712	*CNIH2*	1.34 (1.00-1.80)	5.36×10^{-2}	1.58 (1.34-1.86)	3.56×10^{-8}	0.845
rs4766566	T/C	12q24.12	12	111706877	*CUX2*	1.35 (1.24-1.48)	3.97×10^{-11}	1.51 (1.38-1.65)	4.03×10^{-20}	0.897

*Allele A is risk-associated allele and allele B is non-risk-associated allele.

†Present GWAS (Gout vs. AHUA)

‡Previous GWAS (Gout vs. Normouricemia) with 945 gout cases and 1,213 controls (ref. 25, Nakayama et al., 2017)

§Ratio of two ORs = OR (Gout vs. AHUA) / OR (Gout vs. Normouricemia)

SUA, serum uric acid; Chr, chromosome; GWAS, genome-wide association study; AHUA, asymptomatic hyperuricemia

Supplementary Table S4 The effect of each gout locus on SUA

SNP	Allele* A/B	Locus	Chr	Position	Gene	Japanese Nakatochi et al, 2019 (n=121,745)			European ancestry Köttgen et al, 2013 (n=110,347)		
						RAF‡	Beta† ± SE	P value	RAF‡	Beta† ± SE	P value
Gout loci identified in the present GWAS (Gout vs. AHUA)											
rs7927466	A/G	11q22.1	11	100141763	*CNTN5*	0.962	-0.004±0.010	0.73	1.000	NA	NA
rs9952962	C/T	18q12.1	18	27535568	*MIR302F*	0.483	-0.001±0.004	0.75	0.775	-0.009±0.005	6.48×10^{-2}
rs12980365	A/G	19p12	19	23395317	*ZNF724*	0.968	0.0002±0.011	0.99	0.805	-0.002±0.005	0.70
Gout loci reported in the previous GWAS (Gout vs. Normouricemia)§											
rs1260326	T/C	2p23.3	2	27730940	*GCKR*	0.559	0.036±0.004	7.56×10^{-19}	0.400	0.055±0.004	1.31×10^{-40}
rs1014290	T/C	4p16.1	4	10001861	*SLC2A9*	0.581	0.125±0.004	1.17×10^{-212}	0.692	0.257±0.004	$< 1.00\times10^{-300}$
rs11733284	A/G	4p12	4	48028097	*NIPAL1*	0.306	0.004±0.004	0.35	0.350	0.016±0.004	2.15×10^{-4}
rs3114020	C/T	4q22.1	4	89083666	*ABCG2*	0.751	0.085±0.005	9.12×10^{-78}	0.400	0.071±0.004	2.58×10^{-66}
rs1165176	G/A	6p22.2	6	25830298	*SLC17A1*	0.838	0.052±0.005	1.54×10^{-22}	0.492	0.064±0.004	3.76×10^{-57}
rs11758351	G/T	6p22.2	6	26203910	*HIST1H4E*	0.131	0.023±0.006	1.25×10^{-4}	0.129	-0.011±0.006	5.42×10^{-2}
rs7903456	A/G	10q23.2	10	88919319	*FAM35A*	0.245	0.031±0.005	1.20×10^{-11}	0.775	0.017±0.004	1.82×10^{-4}
rs2285340	A/G	11q13.1	11	64435906	*SLC22A12*	0.179	0.060±0.005	3.54×10^{-30}	0.000	NA	NA
rs4073582	C/T	11q13.2	11	66050712	*CNIH2*	0.923	0.179±0.008	2.25×10^{-122}	NA	NA	NA
rs4766566	T/C	12q24.12	12	111706877	*CUX2*	0.676	0.056±0.004	7.27×10^{-40}	0.260	-0.016±0.005	5.39×10^{-4}

The effect of each locus on SUA as revealed by our recent GWAS meta-analysis of SUA with a total of 121,745 Japanese subjects (ref. 47, Nakatochi et al., 2019), and by GWAS meta-analysis of SUA with a total of 110,347 individuals of European ancestry within the Global Urate Genetics Consortium (GUGC) (ref. 22, Köttgen et al., 2013)

*Allele A is risk-associated allele and allele B is non-risk-associated allele.

†The beta value represents change in z-score per risk allele copy for the SNP.

‡RAF for GUGC was calculated from HapMap phase II r24 CEU samples.

§Gout loci reported in the previous GWAS (Gout vs. Normouricemia) (ref. 25, Nakayama et al., 2017).

SUA, serum uric acid; Chr, chromosome; RAF, risk allele frequency; GWAS, genome-wide association study; AHUA, asymptomatic hyperuricemia; SNP, single nucleotide polymorphism

Supplementary Table S5 The effect of each gout locus from the results of the gout GWAS (Gout vs. Non-gout) using individuals of European ancestry

SNP	Allele A/B*	Locus	Chr	Position	Gene	European ancestry<br>Gout vs. Non-gout†<br>OR‡ (95%CI)	 <br>P value§
Gout loci identified in the present GWAS (Gout vs. AHUA)							
rs7927466	A/G	11q22.1	11	100141763	*CNTN5*	NA	NA
rs9952962	C/T	18q12.1	18	27535568	*MIR302F*	0.92 (0.85-0.99)	3.81×10^{-2}
rs12980365	A/G	19p12	19	23395317	*ZNF724*	1.13 (1.03-1.23)	8.54×10^{-3}

*Allele A is risk-associated allele and allele B is non-risk-associated allele.

†The gout GWAS (Gout vs. Non-gout) of European ancestry with 2,115 gout cases and 67,259 non-gout controls within the Global Urate Genetics Consortium (GUGC) (ref. 22, Köttgen et al., 2013)

‡The odds ratio (OR) value represents increased risk of gout per risk allele copy for the SNP.

§The P value has been corrected for genomic control.

SUA, serum uric acid; Chr, chromosome; GWAS, genome-wide association study; AHUA, asymptomatic hyperuricemia
